# Effect of Benzene Exposure on the Urinary Biomarkers of Nucleic Acid Oxidation in Two Cohorts of Gasoline Pump Attendants

**DOI:** 10.3390/ijerph16010129

**Published:** 2019-01-05

**Authors:** Mariella Carrieri, Daniela Pigini, Andrea Martinelli, Enrico Paci, Federica Maratini, Fabiola Salamon, Giovanna Tranfo

**Affiliations:** 1 Department of Cardiac, Thoracic, Vascular Sciences and Public Health, University of Padova, via Giustiniani, 2, 35128 Padova, Italy; mariella.carrieri@unipd.it (M.C.); andrea.martinelli@unipd.it (A.M.); federica.maratini@gmail.com (F.M.); fabiola.salamon@unipd.it (F.S.); 2 INAIL Research, Department of Occupational Medicine, Epidemiology, Occupational and Environmental Hygiene Via di Fontana Candida 1, 00040 Monte Porzio Catone, Rome, Italy; d.pigini@inail.it (D.P.); e.paci@inail.it (E.P.)

**Keywords:** benzene exposure, SPMA, nucleic acids oxidation, oxidative stress, urinary biomarkers

## Abstract

(1) Background: The oxidized guanine derivatives excreted into urine, products of DNA and RNA oxidation and repair, are used as biomarkers of oxidative damage in humans. This study aims to evaluate oxidative damage in gasoline pump attendants occupationally exposed to benzene. Benzene is contained in the gasoline but it is also produced from traffic and from smoking. (2) Methods: Twenty-nine gasoline pump attendants from two major cities of Saudi Arabia and 102 from Italy were studied for urinary 8-oxo-7,8-dihydroguanine (8-oxoGua), 8-oxo-7,8-dihydro-2′-deoxyguanosine (8-oxodGuo), 8-oxo-7,8-dihydroguanosine (8-oxoGuo), and S-phenyl-mercapturic acid (SPMA) for benzene exposure and urinary cotinine for smoking status assessment by liquid chromatography-tandem mass spectrometry. Airborne benzene was also assessed in the Italian group by gas-chromatography with flame ionization detector (GC-FID). (3) Results: The results suggest that high levels of benzene exposure can cause an accumulation of SPMA and bring about the formation of the oxidation biomarkers studied to saturation. At low exposure levels, SPMA and oxidation biomarker levels were correlated among them and were associated with the smoking habit. (4) Conclusions: The study confirms the association between benzene exposure and the excretion of nucleic acid oxidation biomarkers and enhances the importance of measuring the smoking habit, as it can significantly influence oxidative damage, especially when the exposure levels are low.

## 1. Introduction

Oxidative stress involves oxidative damage to biomolecules, including nucleic acids, lipids, and proteins, and it has been implicated in the normal ageing process and the initiation and progression of several diseases, including neurodegenerative, cardiovascular, and kidney diseases, alterations in the reproductive system, and different types of cancer. In humans, the products of DNA and RNA oxidation are the extracellular oxidized guanine derivatives which are excreted into the urine, and can be used as biomarkers of oxidative damage.

Hydroxyl radical—•OH is mostly generated in cells through the Fenton reaction: the reactivity of •OH is very high with the nucleobases and 2-deoxyribose moieties of DNA by addition to the C8 of purine bases. ^1^O_2_ also reacts with guanine, giving rise predominantly in cellular DNA to 8-oxoGua [1]. 8-oxo-7,8-dihydro-2′-deoxyguanosine (8-oxodGuo) is the oxidized form of the nucleoside deoxyguanosine, formed by guanine attached to a deoxyribose, from the 2′-deoxyribonucleotide pool [2]; neither cell death nor diet contribute considerably to urinary 8-oxodG, and its levels are not influenced by long-term storage of urine specimens at −20 °C [3]. 8-oxo-7,8-dihydroguanosine (8-oxoGuo) is the oxidized form of the nucleoside guanosine that is formed by guanine attached to a ribose in RNA. 8-oxo-7,8-dihydroguanine (8-oxoGua) is the oxidized form of the guanine, coming predominantly from DNA [3,4,5]. The 7,8-dihydro description is used to indicate saturation of the double bond between N7 and C8 atoms of the parent unmodified guanine, from which the damaged nucleobase is derived [1].

The presence of 8-oxoGua residues in DNA leads to GC to TA transversion unless repaired prior to DNA replication. Several DNA repair pathways have evolved that protect organisms subject to oxidative stress from the deleterious effects of DNA damage [6].

Effects of workplace exposure to asbestos [7], benzene [8], fine particulate matter [9], polycyclic aromatic hydrocarbons [10], silica [11], metals [12], styrene [13], toluene, and xylenes [14] on the level of urinary 8-oxodGuo have been reported, with controversial results. There is still a lack of well-established dose-response relations between occupational or environmental exposures and the induction of 8-oxodGuo. Smoking has been identified as a confounding factor but various occupational studies did not reveal higher levels of 8-oxodGuo in smokers. Despite these conflicting results, 8-oxodGuo is reported to be a promising biomarker of oxidative stress associated with chemical exposure [14].

A mismatch of 8-oxo-7,8-dihydroguanine (8-oxoGuo) in RNA to adenine leads to transcriptional errors and produces abnormal protein. The measurement of 8-oxoGuo in urine has potential as a novel means of evaluating the aging process [15].

The advent of liquid chromatography coupled to tandem mass spectrometry (HPLC-MS/MS) has enabled the determination of oxidized guanine derivatives in urine, with reduced sample manipulation and reduced risk of pre-analytical artifacts as compared to blood and tissues [8]; the use of stable isotope internal standardization (isotopic dilution) compensates for analyte loss during sample workup and analysis, and for matrix effects, which makes it the method of choice for these analytes according to Barregard et al. (2013) [16]. However, the analytical and clinical validation of these biomarkers remains a research issue. In particular, the establishment of reference intervals in different populations is a prerequisite for the use of these compounds as clinical screening tools of disease as well as biomarkers for monitoring the early effects of oxidizing agents [17].

The objective of the present study is to determine the levels of urinary 8-oxodGuo, 8-oxoGuo, and 8-oxoGua in two groups of gasoline pump attendants occupationally exposed to benzene, including both smokers and non-smokers, and to explore their relationship with the urine concentrations of S-phenyl mercapturic acid (SPMA), the exposure biomarker for occupational exposure to benzene suggested by the American Conference of Governmental Industrial Hygienists (ACGIH) [18], and the levels of urinary cotinine, a metabolite of nicotine used to assess the smoking status and intensity [19]. Samples were tested by means of a high-performance liquid chromatography-electrospray tandem mass spectrometry (HPLC/MS-MS) analytical method using isotopic dilution for the quantitative analysis.

## 2. Materials and Methods

### 2.1. Study Population

The study sample consisted of two groups of gasoline pump attendants, whose characteristics are summarized in Table 1.

Group 1 subjects performed an extended work shift (10–12 h) under extreme environmental conditions (temperatures around 40 °C), and are therefore exposed to an increased risks of inhalation of gasoline vapors. Smokers are not allowed to smoke during the work shift. Samples were collected before and at the end of the working shift in the period July–August 2014.

Group 2 subjects performed an 8 h work shift. Smokers are allowed to smoke during the work shift. Samples were collected at the end of the working shift in the period May 2012–May 2017.

Each subject was asked to complete a questionnaire for collecting information on age, occupational history, living and smoking habits, hobbies, use of chemical products. The smoking habit was also evaluated by means of urinary concentration of cotinine (smokers ≥ 100 µg/g creatinine). Before providing the urine sample, all gave written informed consent to participation in the study. As our study was considered an observational study on the basis of the definitions of the European Directive 2001/20/EC, the approval of an ethics committee was not requested.

### 2.2. Urine Sample Collection

Urine samples were collected in sterile plastic containers, divided into three aliquots in polypropylene screw-cap tubes in the collection site and then shipped refrigerated to the laboratory where they were stored frozen at −20 °C until analysis. With reference to the stability during storage of the analytes, it was reported that 8-oxodGuo in urine is stable for 24 h at room temperature and for more than 2 years at −80 °C. Poulsen et al. reported stability of 8-oxo-dGuo at −20° for 6 years [20], while Loft et al. for 15 years [21]. No information is reported for the storage of other analytes in urine samples in the literature.

One aliquot of urine sample was used for determination of SPMA and cotinine, one for the creatinine concentration and the third one for the determination of urinary 8-oxoGua, 8-oxodGuo, and 8-oxoGuo.

### 2.3. Chemicals and Supplies

The analytical reference standards of 8-oxoGua, 8-oxodGuo and 8-oxoGuo were purchased by Spectra 2000 s.r.l (Rome, Italy). The deuterium labeled internal standards [^13^C^15^N_2_] 8-oxodGuo and [^13^C^15^N_2_] 8-oxoGuo and DL-SPMA-3,3-d_2,_ were obtained from CDN Isotopes Inc (Pointe-Claire, Quebec, Canada). [^13^C^15^N] 8-oxoGua (98%) was obtained from Cambridge Isotope Laboratories, Inc. (Cambridge, MA, USA).

Glacial acetic acid, 30% NH_3_, dimethyl sulfoxide, sodium hydroxide solution (50–52% in water), and CHROMASOLV^®^ gradient grade (≥99.9% methanol and acetonitrile for HPLC/MS and ≥99.9% carbon disulfide low benzene content) were obtained from Sigma Aldrich (Saint Louis, MO, USA). Purified water was obtained from a Milli-Q Plus system (Millipore, Milford, MA, USA). An Anotop 10LC syringe filter device (0.2 µm pore size, 10-mm diameter) was purchased from Whatman Inc. (Maidstone, UK). The SPE cartridges, Sep-Pak Plus C18 (10 mL, 500 mg) were supplied by Waters. A Sinergi Fusion C18 column (150 × 4.6 mm, 4 μm) supplied by Phenomenex (Phenomenex, Torrance, CA, USA) and Synergi 4U Polar RP C18 column (150 × 4.6 mm, 4 μm) supplied by Phenomenex (USA) were used throughout the study.

The urine samples were analyzed on a Series 200 LC quaternary pump (PerkinElmer, Norwalk, CT, USA), coupled with a AB/Sciex API 4000 triple-quadrupole mass spectrometry detector equipped with a Turbo Ion Spray (TIS) probe.

### 2.4. Analytical Methods

#### 2.4.1. Environmental Monitoring

Exposure to benzene has been assessed in the subjects of Group2 at the breathing zone level, using personal diffusive samplers containing an active carbon cartridge (Radiello^®^). Samplers were worn throughout the 8-h work shift by the exposed subjects. Analysis of benzene from Radiello^®^ was performed by gas chromatography with a flame ion detector (GC-FID) after desorption of benzene from the active carbon with low benzene content carbon disulfide according to the modified NIOSH method [22]. The detection limit of the procedure was 1.5 µg/m^3^.

#### 2.4.2. Biological Monitoring

All analytical determinations were performed by isotopic dilution HPLC-MS/MS. The concentration of the analytes were determined according to the analytical methods previously validated in our laboratories [23]. Briefly, for SPMA analysis the urine samples were treated with HCl 6 N at pH = 2 and then purified on SPE obtaining two fractions, one containing SPMA and one containing cotinine, that were separately analyzed by HPLC-MS/MS. The precursor→product ionic transitions monitored were, in the negative ion mode 238.1→109.1 for SPMA and 240.1→109.1 for SPMAd^2^, and in the positive ion mode 177.3→80.10 for cotinine and 180.3→80.10 for cotinine-d^3^. For the determination of the three biomarkers of nucleic acid oxidation the urine samples were centrifuged and analyzed by HPLC-MS/MS. The precursor→product ionic transitions monitored were 168.0→140.0 and 171.0→143.0 for 8-oxoGua and its internal standard ([^13^C^15^N_2_] 8-oxoGua), 284.3→168.0 and 287.13→171.1 for 8-oxodGuo and its internal standard ([^13^C^15^N_2_] 8-oxodGuo), and 300.24→168.2 and 303.24→171.0 for 8-oxoGuo and its internal standard ([^13^C^15^N_2_] 8-oxoGuo), respectively. The 1.5 version of Analyst^®^ software was employed for instrument control.

The final concentration of the analytes is expressed in µg/g of creatinine, to normalize values with respect to urine dilution variability. Urinary creatinine has been determined by the method of Jaffè [24], using alkaline picrate test with UV/VIS detection at 490 nm.

The following table (Table 2) summarizes the concentration ranges and the limits of quantitation (LOQ), calculated using the approach based on the standard deviation of the response and the slope, for all the analytes.

### 2.5. Statistical Analysis

Statistical analysis was carried out using the StatsDirect statistical software (Statsdirect 3.1.18 version, Statsdirect Ltd., Cambridge, UK). Differences between smokers and non-smokers within groups were assessed using the Mann–Whitney U-test. The difference between the urinary parameters measured before and after the work shift within Group 1, both for all subjects or subdivided according to the smoking habit, was assessed using the paired *t*-test (*p* >0.05).

Pearson’s log correlation was used to analyze the correlation between the urinary parameters. Multivariate analysis was also employed to evaluate the influence of different variables on oxidation biomarkers excretion. In all tests, a *p*-value lower than 0.05 (two-tailed) was considered as statistically significant.

## 3. Results

The metabolite concentrations for the chemical exposure (SPMA and cotinine) and the oxidation biomarkers (8-oxoGua, 8-oxodGuo and 8-oxoGuo) are reported in Table 3 for Group 1 and in Table 4 for Group 2.

The urinary concentrations were normalized to the creatinine concentration and the mean, standard deviation (SD), and the 5th, 50th (corresponding to the median value), and 95th percentiles are reported.

SPMA values are very high in all subjects of Group 1 (smokers and non-smokers), with the 95th percentile being even higher or equal to the ACGIH limit of 25 µg/g creatinine that indicates a high occupational exposure to benzene. The SPMA levels were also high both before and after the work shift: possible explanations for this result is the extended working shift (10–12 h) that could lead to a probable accumulation of the benzene metabolite. In fact as the SPMA half-life is about nine hours, it continues to be excreted until the beginning of the following shift never reaching the background level. This hypothesis is confirmed by the fact that before the working shift also non-smokers present high values for urinary SPMA. There are also no statistically significant differences for SPMA between smokers and non-smokers (before shift *p* = 0.504, post shift *p* = 0.134, Mann–Whitney test), probably due to the very high occupational benzene exposure levels that cover the contribution of smoking, while cotinine values of smokers are higher than non-smoker values (*p* <0.0001, Mann–Whitney test).

With reference to the oxidation biomarkers, no differences were found, for all three variables, between smokers and non-smokers (*p* > 0.05, Mann–Whitney test) or between before and after the work shift, both in all subjects and subdivided according with the smoking habit (*p* > 0.05, paired *t*-test).

The subjects of Group 2 were exposed to benzene levels in range <1.5–80 µg/m^3^ (mean 15 µg/m^3^, median 7 µg/m^3^). These exposure levels are very low, only slightly higher than the limit value of air quality adopted in Italy for the annual concentration of benzene equal to 5 µg/m^3^ [25] and lower than those found in a previous study carried out on gasoline station attendants [26].

In this group we found much lower values of SPMA than in Group 1, comparable to those measured in another study carried out using the same analytical method in subjects non-occupationally exposed to benzene [27]. A statistically significant difference was found between smokers and non-smokers: for SPMA *p* < 0.0001 and for 8-oxoGua *p* = 0.016 (Mann–Whitney test) but not for the other two oxidation biomarkers. In Group 2, because of the lower benzene exposure, the smoking contribute becomes more evident. These results partly confirm what found in a previous study carried out on 131 general population volunteers non-professionally exposed to benzene [21]. The 8-oxoGua was also significantly higher in women than in men (*p* = 0.027) were there was a higher percentage of smokers (51.52% vs. 39.13%, data not shown).

In Group 1, 8-oxo-Gua is statistically significant higher before the workshift than after, only in non-smokers, while in Group 2 is higher in smokers than in non-smokers (after the workshift). This is the only discriminant parameter within the groups in the nucleic acid oxidation biomarkers, and we do not have an explanation for this; 8-oxoGua seems to be more variable and difficult to interpret as it is the resultant of two different effects, both on RNA and on DNA.

The comparison between the two groups showed highly statistically significant differences for all analytes, that are higher in Group 1 than in Group 2: SPMA (*p* < 0.0001), cotinine (*p* = 0.0003), 8-oxoGua (*p* = 0.0097), 8-oxoGuo (*p* < 0.0001) and 8-oxodGuo (*p* < 0.0001).

The log-correlations among the different analytes were also studied in the two groups (Table 5 and Table 6). In Group 1 results before and after shift have been analyzed together as we found no significant differences between them. A statistically significant correlation was found within the three oxidation biomarkers but not between them and SPMA; besides no correlation was found between SPMA and cotinine in smokers (for which benzene exposure is predominant with respect to smoking) confirming that they are not coming from the same source (data not shown).

Both in smokers and in non-smokers there were very low correlations between SPMA and nucleic acid oxidation products. This observation, together with the very high values of 8-oxodGuo and 8-oxoGuo, that are correlated among them, suggests that these biomarkers could have reached a constant value (plateau) due to the very high benzene exposure.

In Group 2, a statistically significant correlation was found within the three oxidation biomarkers and between SPMA and 8-oxoGua.

In this case the three oxidation biomarkers and SPMA were also significantly correlated with the cotinine in smokers, showing that nucleic acid oxidation is actually correlated with active smoking, that contains benzene but also other toxicants, not determined in this study, confirming the results of Tranfo et al. (2017) [22].

In non-smokers the only significant correlation was found between 8-oxodGuo and 8-oxoGuo, as also in smokers and in Group 1.

A subsequent multivariate analysis showed that job seniority was the only variable among others (age, body mass index, airborne benzene exposure) that negatively influences the excretion of both 8-oxodGuo and 8-oxoGuo (*p* = 0.0355 and *p* = 0.0011, respectively). This result can be justified by the fact that a longer experience in the task lead to a major risk perception and attention. The result is in disagreement with a recent study on gasoline station attendants that showed a positive correlation between 8-oxodGuo measured by ELISA test and low benzene exposure (measured by means of biomonitoring of its metabolite urinary trans, trans muconic acid), age, and job seniority [28].

## 4. Conclusions

In the present study the occupational exposure to benzene of the two groups of gasoline station attendants were very different and provided different information on the aim of the study. The benzene exposure of Group 1, assessed by biological monitoring, was significantly higher than that found in Group 2 assessed by environmental and biological monitoring. In the first group the results suggest that very high levels of benzene exposure can cause a saturation of SPMA metabolism so that the smoking effect is masked, while in Group 2 the statistically significant difference in cotinine levels between smokers and non-smokers is reflected in the SPMA levels.

The results found in Group 2 showed that benzene exposure levels are very low and comparable to the exposure of the general population not occupationally exposed to benzene. The SPMA and oxidation biomarkers levels were correlated among them and associated with the smoking habit, whose effect, at these exposure levels, is more evident. This enhances the importance of measuring the smoking habit of the subjects, which can significantly influence oxidative damage, especially when the exposure levels are low.

The comparison between the two groups showed highly statistically significant differences both for the benzene metabolite and the nucleic acid oxidation biomarkers, that are higher in Group 1 than in Group 2, confirming the association between benzene exposure and the measured oxidation biomarkers.

## Figures and Tables

**Table 1:** Characteristics of the two groups studied.

Gasoline Pump Attendants.	Location	*n*	Smokers (%)	Age	Males	Females	Urine Sampling
**Group 1**	Saudi Arabia	29	14 (48.28)	20–45	29	0	Before and after shift
**Group 2**	Italy	102	44 (43.14)	22–63	69	33	After shift

**Table 2:** Concentration ranges and quantitation limits for the analytes. SPMA: S-phenyl-mercapturic acid.

Metabolite	SPMA	Cotinine	8-oxoGua	8-oxodGuo	8-oxoGuo
Concentration range µg/L	0–25	0–2500	0–915.89	0–22.66	0–74.81
Limit of quantitation (LOQ) µg/L	0.08	37.61	0.91	0.07	0.05

**Table 3:** Concentrations (µg/g creatinine) of metabolites for the chemical exposure (SPMA and cotinine) and the oxidation biomarkers (8-oxoGua, 8-oxodGuo and 8-oxoGuo) in Group 1, before and after shift, stratified by smoking habit.

**Before Shift**								
**Analyte**	**Smokers (*n* = 14)**				**Non-Smokers (*n* = 15)**			
	**Mean (SD)**	**Percentile**			**Mean (SD)**	**Percentile**		
		**5th**	**50th**	**95th**		**5th**	**50th**	**95th**
**SPMA**	13.97 (12.93)	2.57	8.52	37.42	14.45 (17.72)	0.58	5.58	49.57
**Cotinine**	1619.97 (1057.26)	380.58	1362.20 *	3203.78	28.71 (28.14)	5.14	19.62 *	87.36
**8-oxoGua**	140.71 (231.25)	2.20	44.66	610.10	81.39 (74.11)	13.10	55.94	231.54
**8-oxodGuo**	15.70 (6.12)	7.62	15.87	24.89	16.47 (5.87)	7.37	17.12	24.75
**8-oxoGuo**	33.32 (17.83)	9.80	30.98	62.12	32.35 (10.03)	20.53	28.86	52.46
**After Shift**								
**Analyte**	**Smokers (*n* = 14)**				**Non-Smokers (*n* = 15)**			
	**Mean (SD)**	**Percentile**			**Mean (SD)**	**Percentile**		
		**5th**	**50th**	**95th**		**5th**	**50th**	**95th**
**SPMA**	14.39 (11.00)	2.42	11.36	34.76	9.89 (9.29)	0.89	6.50	24.73
**Cotinine**	1404.18 (908.34)	514.09	1353.53 *	2631.33	17.64 (16.90)	3.20	11.27 *	47.37
**8-oxoGua**	72.95 (78.89)	1.44	48.96	207.02	40.03 (41.88)	2.45	21.44	120.00
**8-oxodGuo**	17.46 (11.54)	7.78	14.46	36.49	12.99 (5.62)	5.05	13.72	21.06
**8-oxoGuo**	31.26 (16.59)	8.68	27.01	61.15	27.19 (15.13)	7.55	26.97	49.53

* smokers vs. non-smokers (Mann-Whitney test).

**Table 4:** Concentrations (µg/g creatinine) of metabolites for chemical exposure (SPMA and cotinine) and oxidation biomarkers (8-oxoGua, 8-oxodGuo and 8-oxoGuo) in Group 2, after shift, stratified by smoking habit.

Analyte	Smokers (*n* = 44)				Non-Smokers (*n* = 58)			
	Mean (SD)	Percentile			Mean (SD)	Percentile		
		5th	50th	95th		5th	50th	95th
**SPMA**	1.09 (0.86)	0.24	0.81 *	3.00	0.14 (0.11)	0.02	0.11 *	0.34
**Cotinine**	623.70 (334.90)	193.36	514.82 **	1269.97	10.88 (16.38)	2.02	4.53 **	47.17
**8-oxoGua**	97.86 (67.36)	26.04	73.94 ***	222.42	69.67 (53.08)	15.87	49.84 ***	194.18
**8-oxodGuo**	4.34 (1.93)	1.68	3.82	7.83	3.86 (1.48)	1.97	3.91	6.23
**8-oxoGuo**	9.70 (5.06)	3.99	8.22	18.68	11.34 (5.83)	4.71	9.92	20.50

* smokers vs. non-smokers (Mann–Whitney test). ** smokers vs. non-smokers (Mann-Whitney test). *** smokers vs. non-smokers (Mann–Whitney test).

**Table 5:** Pearson’s log-correlations in Group 1.

**Smokers**			
	**8-oxodGuo**	**8-oxoGuo**	**SPMA**
8-oxoGua	0.44 *p* = 0.018	0.70 *p* < 0.0001	0.14 ns
8-oxodGuo		0.78 *p* < 0.0001	0.19 ns
8-oxoGuo			0.27 ns
**Non-Smokers**			
	**8-oxodGuo**	**8-oxoGuo**	**SPMA**
8-oxoGua	0.26 ns	0.38 *p* = 0.039	0.04 ns
8-oxodGuo		0.81 *p* < 0.0001	0.28 ns
8-oxoGuo			0.20 ns

**Table 6:** Pearson’s log-correlations in Group 2.

**Smokers**				
	**8-oxodGuo**	**8-oxoGuo**	**SPMA**	**Cotinine**
8-oxoGua	0.32 *p* = 0.03	0.07 ns	0.40 *p* = 0.007	0.43 *p* = 0.003
8-oxodGuo		0.64 *p* < 0.0001	0.27 ns	0.34 *p* = 0.02
8-oxoGuo			0.24 ns	0.34 *p* = 0.02
SPMA				0.75 *p* < 0.0001
**Non-Smokers**				
	**8-oxodGuo**	**8-oxoGuo**	**SPMA**	**Cotinine**
8-oxoGua	0.09 ns	−0.15 ns	0.009 ns	0.21 ns
8-oxodGuo		0.40 *p* = 0.002	−0.002 ns	0.03 ns
8-oxoGuo			0.03 ns	0.17 ns
SPMA				0.20 ns
